# Creating a Digital Psychoeducation Programme for bipolar disorder in the COVID-19 pandemic

**DOI:** 10.1192/j.eurpsy.2022.1458

**Published:** 2022-09-01

**Authors:** R. Gadelrab, S. Simblett, J. Hook, S. Rickwood, J. Martinez, M. Johnstone, C. Flower, S. Bourne, A. Young, K. Macritchie

**Affiliations:** 1 Institute Of Psychiatry, Psychology & Neuroscience, King’s College London, Centre For Affective Disorders, London, United Kingdom; 2 Institute of Psychiatry, Psychology & Neuroscience, King’s College London, Psychology, London, United Kingdom; 3 South London and Maudsley NHS Foundation Trust, National Affective Disorder Service, london, United Kingdom

**Keywords:** digital, BIPOLAR, psychoeducation, covid

## Abstract

**Introduction:**

The Covid-19 pandemic profoundly affected delivery and accessibility of mental health care services at a time when most needed. The OPTIMA Mood Disorder Service, a specialist bipolar disorder service, adapted group psychoeducation programme for delivery on-line.

**Objectives:**

We report the feasibility of creating a digital psychoeducation programme.

**Methods:**

The OPTIMA ten session group psychoeducation programme was converted into a ‘Digital’ intervention using video-conferencing. Sessions offered a range of key topics, derived from the initial Barcelona Group Psychoeducation Programme. At the time of writing, OPTIMA had fully completed two 10 session digital courses.

**Results:**

A total of 12 people (6 in each group) consented to be part of a service evaluation of the digital groups. Just over half of the participants were women (7/12; 58.3%) and one identified as being non-binary (8.3); remaining participants were men. Age of participants ranged from 25 years to 65 years (Mean=42.3; SD=13.1). Data showed a high level of engagement (77%) All participants reported some improvement with a mean Bipolar Self-Efficacy scale (BPSES) post-group score of 105.6 (SD=14.8). At group level, this change was not statistically significant (F (1, 15) = 0.71, p=0.41). At an individual level, two out of five showed a reliable change index >1.96.

**Conclusions:**

Delivering a ‘digital’ group psychoeducation programme was possible due to careful planning and programme development. There was good uptake from service users suggesting it is a feasible approach with preliminary evidence of clinical benefit.

**Disclosure:**

No significant relationships.

